# Luminescent Properties of Surface Functionalized BaTiO_3_ Embedded in Poly(methyl methacrylate)

**DOI:** 10.3390/ma7010471

**Published:** 2014-01-16

**Authors:** Sebastian Requena, Srijan Lacoul, Yuri M. Strzhemechny

**Affiliations:** Department of Physics and Astronomy, Texas Christian University, TCU Box 298840, Fort Worth, TX 76129, USA; E-Mails: s.lacoul1@tcu.edu (S.L.); y.strzhemechny@tcu.edu (Y.M.S.)

**Keywords:** BaTiO_3_, BTO, polymethyl methacrylate, PMMA, photoluminescence, inorganic-organic nanocomposite

## Abstract

As-received BaTiO_3_ nanopowders of average grain sizes 50 nm and 100 nm were functionalized by (3-aminopropyl)triethoxysilane (APTES) and mixed with poly(methyl methacrylate)/toluene solution. The nanocomposite solution was spin coated on Si substrates to form thin films. The photoluminescence spectrum of the pure powder was composed of a bandgap emission at 3.0 eV and multiple bands centered about 2.5 eV. Surface functionalization of the BaTiO_3_ powder via APTES increases overall luminescence at room temperature while only enhancing bandgap emission at low-temperature. Polymer coating of the functionalized nanoparticles significantly enhances bandgap emissions while decreasing emissions associated with near-surface lattice distortions at 2.5 eV.

## 1. Introduction

Barium titanate (BTO)/poly(methyl) methacrylate (PMMA) nanocomposites have recently been gaining popularity due to their auspicious properties desirable in optoelectronic applications. In particular, BTO’s high index of refraction and high dielectric constant at room temperature show great promise in the fabrication of working high refractive index BTO/PMMA optics for the terahertz regime [1] and development of high capacitance BTO/polymer capacitors [2,3]. Synthesis of high quality BTO/PMMA nanocomposites has been accomplished via several different techniques: solution-mixing [4–6], emulsion polymerization [3,7,8], and melt-mixing [1]. Many properties of these materials were reported in detail. However, luminescent properties of BTO/PMMA nanocomposites are largely unreported. In particular, there is no substantial understanding of the role the BTO/PMMA interface plays in possible changes in the luminescence of the composite compared to that of the constituent matrix and filler.

BTO itself has a complex photoluminescent spectrum. This material has a perovskite structure with a direct bandgap of 2.8–3.2 eV, which has been shown to be tunable to 3.47 eV with decreasing particle size due to the quantum confinement effects [9]. It was found that many radiative transitions in BTO manifest themselves only in nanoscale crystals. For example, multiple bands in the visible part of the spectrum were observed in very thin films and nanoparticles (NPs) [10–12]. In the work by Zhang *et al*. [13], the authors showed that the intensity of the visible emission bands increases with decreasing particle sizes. It was suggested that surface states and crystal lattice defects acting as optical absorption centers could be responsible for the observed behavior. Visible luminescent features have been attributed to the recombination of localized excitons within the disordered BTO octahedron, due to the Ti *d*-orbital surface states in the forbidden gap [10,14]. The emerging consensus suggests that both the structural disorder and the surface play a critical role in the photoluminescence spectrum of nanoscale BTO.

On the other hand, PMMA is an outstanding matrix material for many oxide fillers, including BTO. PMMA is strong, light, and transparent to light with wavelengths below ~300 nm. While ideal perfectly polymerized PMMA should not have any substantial luminescence, it is well known that unsaturated ketone/aldehyde groups do form in PMMA and can act as optical centers at 2.9 eV [15,16]. PMMA’s properties are well understood and easily modified by doping with various copolymers and dye molecules. PMMA is also biocompatible.

It is well established that in many instances luminescent spectra of oxide/polymer composites do not represent a simple superposition of the luminescent features of a matrix and a filler, but they can also reveal many processes germane to the composite nature of the material, such as modified scattering [1], random lasing, and additional emissions [17] occurring at the matrix/filler interface. The latter is of particular interest since numerous characteristics of the oxide fillers are dominated by surface properties due to the high surface-to-volume ratio of nanoparticles. For example, photoluminescence (PL) studies of ZnO/PMMA nanocomposites have shown that embedding ZnO nanoparticles in PMMA quenches defect-related emissions while enhancing excitonic luminescent processes attributable to the interface between ZnO and the polymer [18–20]. This discovery has since been implemented to improve device efficiency in ZnO/PMMA-based photodetectors [21]. Ability to control and modify BTO surface properties in such composites is desirable and may lead to improved performance and new applications for BTO/PMMA as well as other nanocomposites. In this paper we investigate photoluminescent properties of BTO/PMMA thin film nanocomposites to elucidate the role of the BTO/PMMA interface in the luminescent characteristics of the composite.

## 2. Experimental Section

### 2.1. Sample Preparation

BTO nanopowders with average particle sizes of 50 nm and 100 nm, hereinafter referred to as BTO50 and BTO100 respectively, were purchased from Inframat Advanced Nanomaterials (Manchester, CT, USA). 3-aminopropyl triethoxysliane (APTES) from Sigma Aldrich (St. Louis, MO, USA) was utilized as a surfactant to minimize aggregation of the nanoparticles and enhance their dispersion in solution. Research grade toluene was mixed with a sufficient amount of APTES to obtain an 8% concentration (*w*/*w*). The as-received NPs were added to the toluene/APTES solution and then reacted at 80 °C for 24 h. The NPs were then centrifuged and recovered from solution. They were washed twice with ethanol to remove any unreacted APTES. PMMA spheres (Sigma Aldrich, St. Louis, MO, USA) of medium molecular weight were added to the toluene/NP solution by 5 wt% of the toluene. The amount of NPs was 30% by volume of the PMMA. It has been shown that when the percent volume of the nanoparticles is greater than 25% of a polymer composite it minimizes the aggregation of the nanoparticles [4]. The solution was then stirred and heated at 60 °C for 1 h with subsequent ultrasonication for 1 h to further reduce formation of aggregates. The solution was spin coated on Si substrates at speeds of 1000 rpm using a SCS6800 NP spin coater to prepare thin films with a nominal thickness of ~600 nm as determined by thin film interference. Two types of composite samples were investigated: containing BTO50 and BTO100 powders. The nanocomposites will be referred to as C50 and C100, respectively. Additionally, a BTO50/PMMA composite was prepared following the above procedure without the APTES modification step. Pure PMMA thin films of equivalent thicknesses were also spin coated following [22] for background PL characterization. All films were cured in air at 100 °C for 1 h to ensure any remaining solvent evaporated.

### 2.2. Characterization

Fourier Transform Interferometry (FTIR) was performed to characterize the nanoparticles before and after functionalization by the APTES. FTIR spectra were obtained by a Thermo Nicolet Nexus 870 FT-IR E.S.P. (Waltham, MA, USA). The samples were pressed into pellets using spectroscopic grade KBr. Baseline corrections were made to all spectra using a blank KBr pellet as background.

PL spectroscopy experiments utilized a CW Kimmon IK5452R-E HeCd laser (Kimmon Koha, Tokyo, Japan) with a wavelength of 325 nm to excite the signal. The spectra were probed by a Spex 1401 monochromator (Horiba Scientific, Edison, NJ, USA) with a spectral resolution of 0.18 cm^−1^ and an RCA C31034 photomultiplier tube detector (Hofstra Group, Santa Fe, NM, USA). A variable frequency chopper provided a reference frequency to a Stanford Research-830 lock-in amplifier for background noise reduction. The samples were mounted inside an evacuated Janis CCS-150 cryostat (Janis, Woburn, MA, USA) operating within a controllable 8–325 K temperature range. Pure powders were studied by packing them into a specially designed sample holder. PL experiments were performed at room temperature and 8 K. Neutral density filters were employed to minimize photobleaching of the composite samples. Incident pump intensity was 0.5 W/cm^2^.

Atomic Force Microscopy (AFM) was used to study the surface roughness of the thin films as well as gain insight regarding the dispersion of NPs in the polymer films. A nano SOLVER NT-MDT AFM instrument (NT-MDT Co., Zelenograd, Moscow, Russia) in tapping mode was used to obtain height profiles of the films. The pure PMMA film, BTO50 in PMMA, and BTO50 modified with APTES in PMMA films were studied.

## 3. Results and Discussion

**Figure 1.**
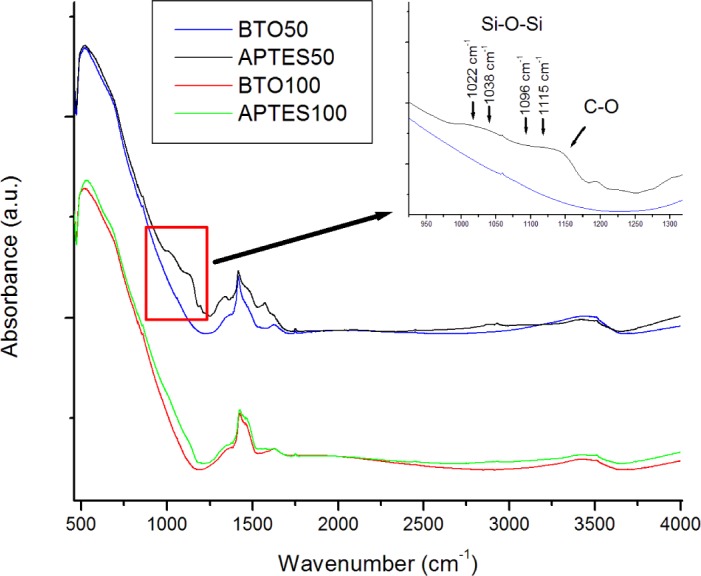
FTIR spectra comparing pure Barium titanate (BTO) powder and (3-aminopropyl)triethoxysilane (APTES) functionalized BTO. Inset is of distinct chemisorbed and physisorbed stretching modes of APTES.

**Figure 2.**
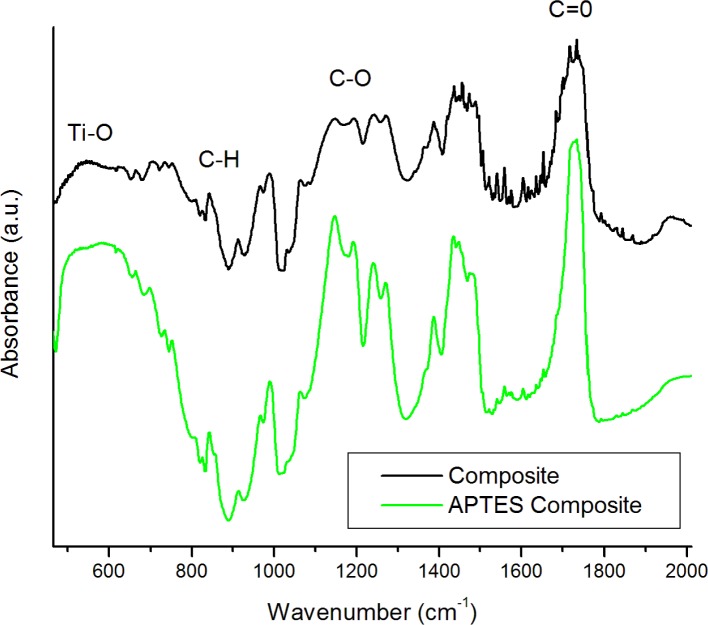
A comparison of FTIR spectra of the BTO50/poly(methyl) methacrylate (PMMA) composite to the APTES modified BTO50/PMMA composite.

FTIR was utilized to confirm the functionalization of the BTO by APTES. The spectra (Figure 1) of the pure powders show a strong absorption band about 578 cm^−1^ assigned to the Ti–O stretching mode characteristic of BaTiO_3_. Some OH stretching modes at 3400 cm^−1^ are attributed to surface hydroxyl groups and possible adsorbed water on the surface of the nanoparticles. The OH peak is rather broad, which is typical due to multiple possible lattice sites on the surface which hydroxyl groups may be situated. The nanoparticles were re-examined by FTIR post-functionalization by APTES. Several notable differences between the spectra are observed. A distinct CH mode at 2975 cm^−1^ is apparent as well as C–O stretching modes between 1000 and 1200 cm^−1^. Si–O–Si modes are (Figure 1 inset) also distributed at 1096, 1022, 1038, and 1115 cm^−1^ confirming the presence of both chemisorbed and physisorbed APTES at the surface of the BTO nanoparticles [23]. An increase in the absorption at 1500–1600 cm^−1^ is also observed which is attributed to amino groups from APTES. The differences in functionalized FTIR spectra are much more apparent in the 50 nm particles, which are expected due to the relative increase in surface functionalized area when compared to the 100 nm particles. Figure 2 shows FTIR spectra of the BTO50/PMMA composite and BTO50/PMMA APTES modified composite. Both spectra contain a sharp peak at 1752 cm^−1^ due to the formation of carboxylates at the surface of the BTO nanoparticles. The ratio of the C=O stretching mode to the Ti–O mode is much larger for the unmodified BTO nanoparticles which is expected due to increased carboxylate formation at the unmodified nanoparticle surface. The modes at 1202 and 1160 cm^−1^ are assigned to ester bond C–O stretching [8].

**Figure 3.**
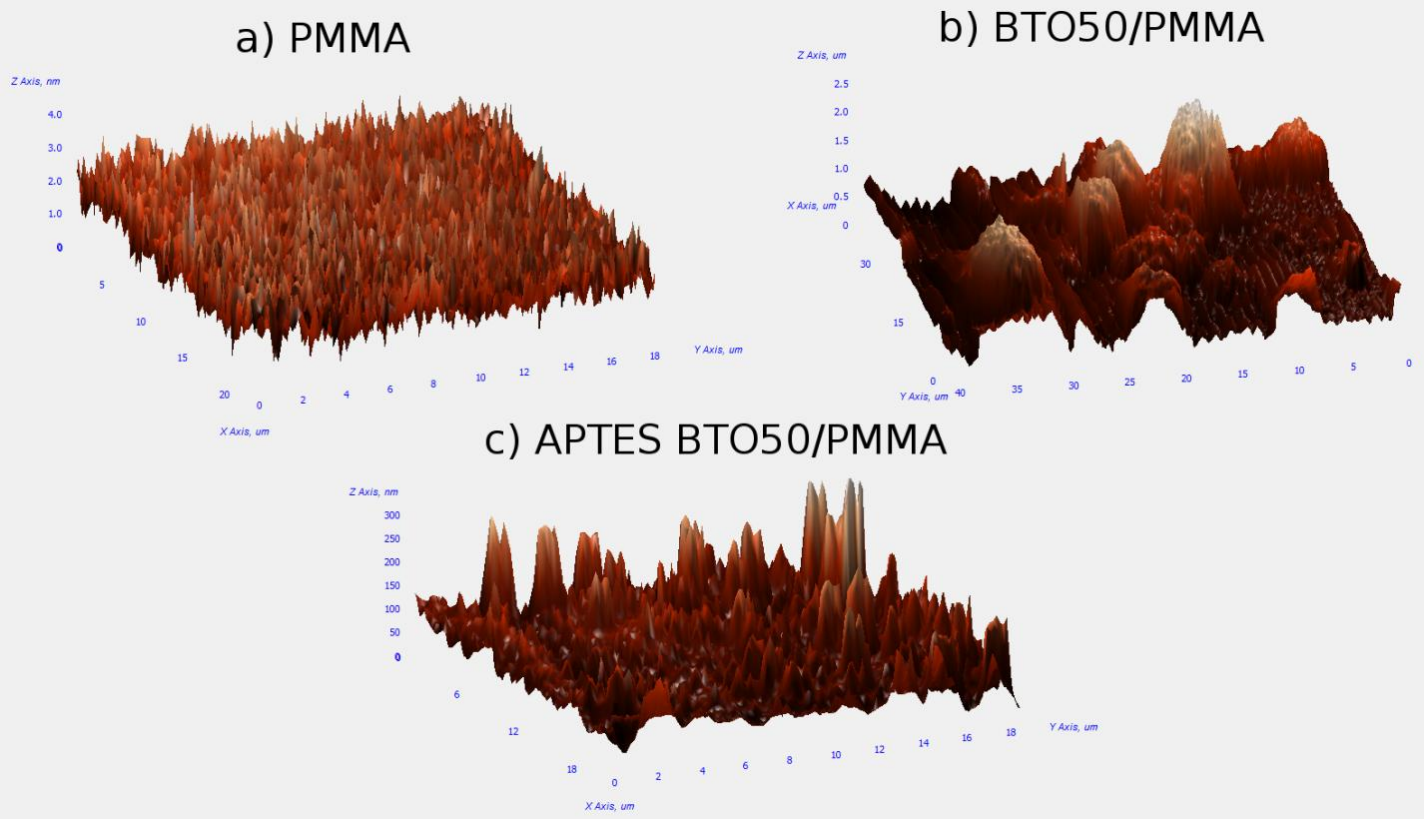
Height profiles of (**a**) pure PMMA films; (**b**) BTO50/PMMA composite without APTES modification step; (**c**) BTO50/PMMA with APTES modification.

AFM analysis of the thin films provided information on the surface roughness as well as whether large agglomerates were formed in the films. Figure 3a is of the pure PMMA film which is relatively flat as per our expectations with an average surface roughness of 0.38 nm. Figure 3b is of the BTO50 in PMMA composite without the use of a surfactant. Large mounds are observed which are due to the formation of aggregates. Analysis of the AFM image shows the aggregates have an average diameter of ~5 μm and the film has an average surface roughness of 0.32 μm. The film of APTES-modified BTO50 particles in PMMA in Figure 3c has a significant reduction in aggregate formation and size with an average surface roughness of 23 nm.

**Figure 4.**
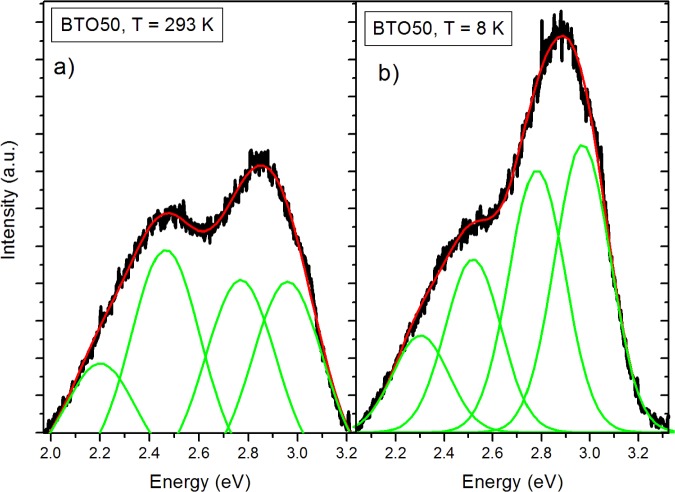
Fitted photoluminescence (PL) spectrum of BTO50 at 293 K (**a**) and 8 K (**b**) showing bandgap of 3.0 eV and the complex visible band composed of emissions at 2.2, 2.5 and 2.7 eV.

**Figure 5.**
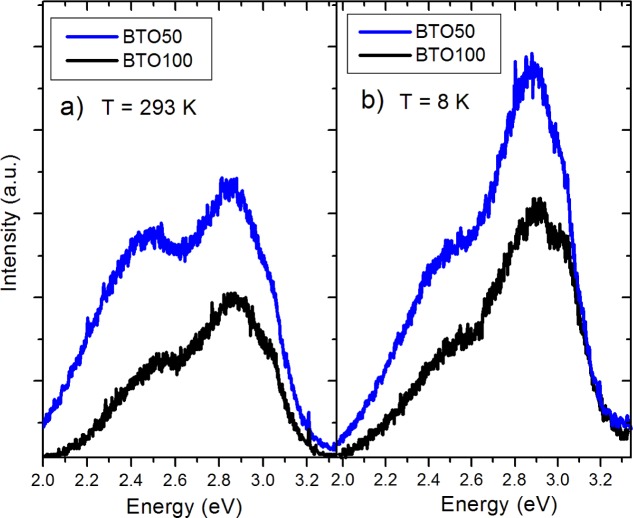
(**a**) Comparison of BTO50 and BTO 100 PL spectra at room temperature; (**b**) Comparison at 8 K. Note that smaller particles have greater luminescence.

The pure BTO50 and BTO100 powders were examined by PL at room temperature and 8 K. Figure 4 shows the fitted PL spectrum of the BTO50 at room temperature (Figure 4a) and 8 K (Figure 4b). All spectra were fitted using multiple Gaussian peaks. The bandgap emission at 3.0 eV (0.28 eV FWHM) is in good agreement with previously reported results [12]. The complex band at ~2.5 eV is composed of three peaks at 2.7 (0.27 eV FWHM), 2.5 (0.27 eV FWHM), and 2.2 eV (0.28 eV FWHM). All fits had a minimum R^2^ = 0.99. The complex band has been fitted by three peaks by [10,11,24]. Our peak energies of the complex band are in good agreement with results by Aguilar *et al*. [24]. This band has been shown to originate from the near-surface disordered TiO_6_ octahedron, which causes splitting of the Ti 3*d* orbitals [10,14]. In Figure 5, the BTO50 and BTO100 samples are compared. At 8 K (Figure 5b), the intensity of the bandgap luminescence is significantly stronger in both the BTO50 and BTO100 samples, while the defect emission doesn’t change appreciably. Lu *et al*. [12] reported the broad visible emission intensity to decrease with increasing temperature and quench completely at 74 K. Our samples show no significant relationship between temperature and PL intensity of the peaks about 2.5 eV. In both BTO50 and BTO100, a very slight blue shift of 0.01 eV of the bandgap is observed at 8 K and the 2.2 eV component of the complex peak blue shifts to 2.3 eV. Other peak energies show no appreciable change. It is consistent with results observed by Lu *et al*. [12], where no significant blue shift was observed with decreasing temperature of the peak energies. The luminescence intensity of the BTO50 powder was greater in magnitude as compared to the BTO100 powder, especially the broad 2.5 eV peak which is in agreement with previous work and supports the idea that surface states are responsible for the visible emissions contribution.

**Figure 6.**
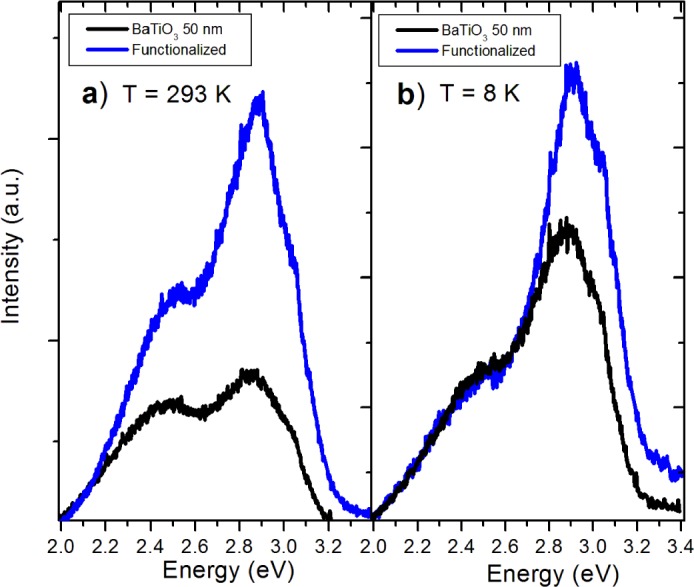
(**a**) Comparison of PL spectra of pure BTO50 powder with APTES functionalized powder; (**b**) Comparison at 8 K.

The surface functionalized nanoparticles were studied with PL prior to embedding in the PMMA. Luminescent emissions of the APTES-modified BTO powders increase across the spectrum at room temperature (Figure 6a). However, the 2.5 eV peak actually quenches to levels comparable to the pure powders at 8 K (Figure 6b). This behavior is observed in nanoparticles of both sizes. The increase of luminescence is believed to be due to the passivation of surface hydroxyl groups which act as non-radiative channels of recombination. The reason for 2.5 eV quenching of the APTES functionalized nanoparticles at low temperature is still unclear.

To analyze the effects of BTO/PMMA mixing on the resulting luminescence spectra, we first corrected the raw PL spectra of the nanocomposites to account for attenuation due to scattering using the following approximation [25]:

$$\frac{I}{I_{o}}=\text{exp}\;4\frac{\pi^{4}}{\lambda^{4}}\left[\frac{n_{p}^{2}-n_{m}^{2}}{n_{p}^{2}+2n_{m}^{2}}\right]d^{3}C_{v}l$$ (1)

**Figure 7.**
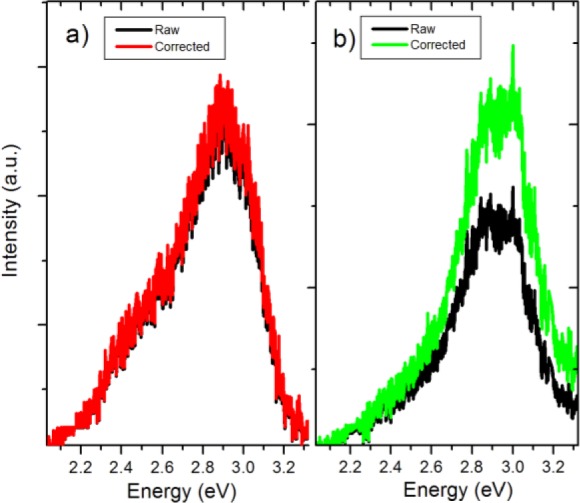
Raw and attenuation corrected PL spectra of (**a**) C50 nanocomposite; and (**b**) C100 nanocomposite.

Here λ is the luminescence wavelength, *n*_p_—refractive index of the filler, *n*_m_—refractive index of the matrix, *d*—diameter of a nanoparticle, *C*_v_—volume fraction of the filler particles in the matrix, and *l*—sample thickness. Figure 7 compares raw and attenuation corrected PL spectra for the C50 and C100 BTO/PMMA nanocomposites. The effects of scattering do not appear to be significant in most of the spectral range.

**Figure 8.**
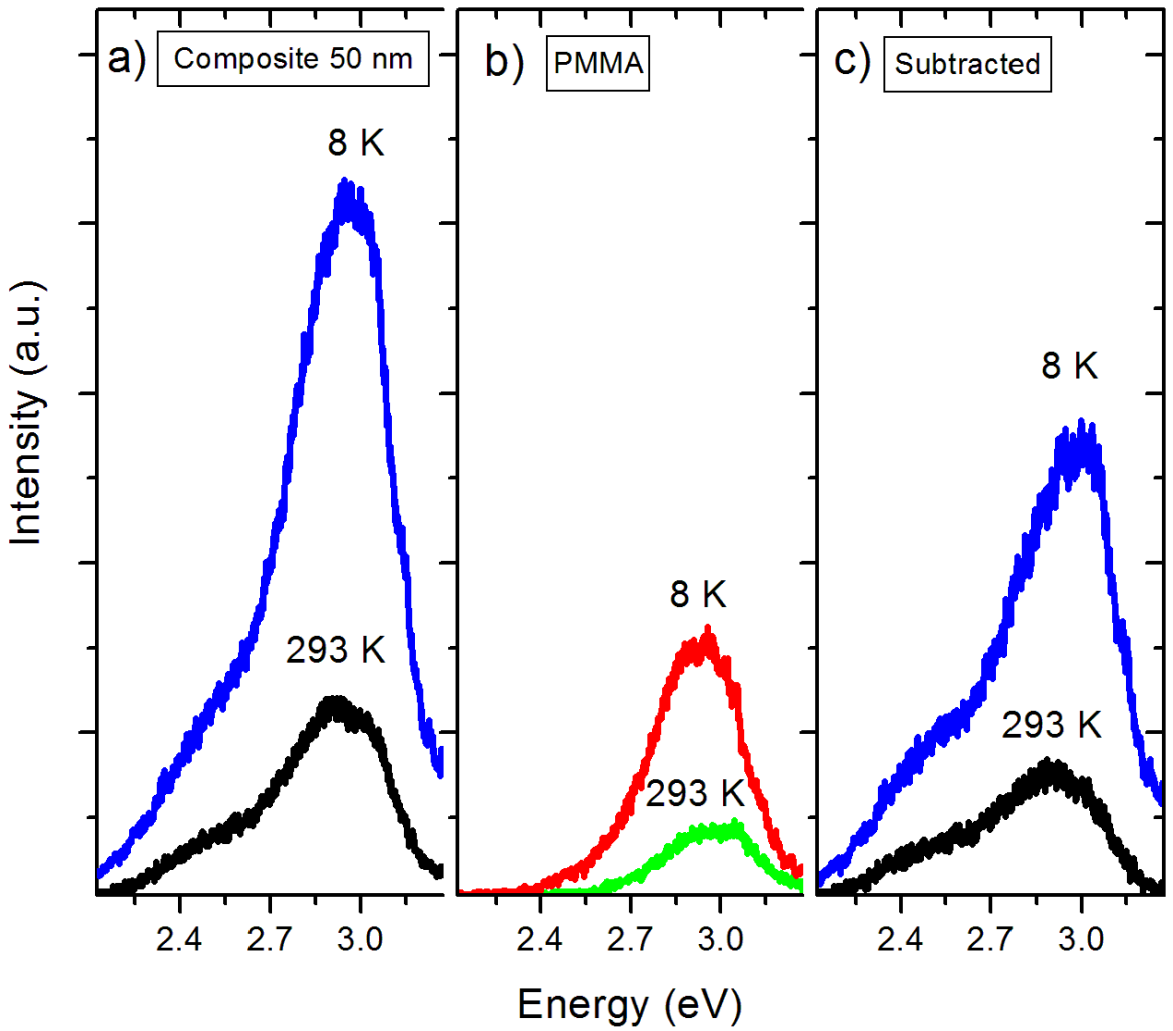
PL spectra of (**a**) Nanocomposite film; (**b**) PL of pure PMMA film of equivalent thickness; (**c**) Nanocomposite with PMMA contributions subtracted.

As the next step in the analysis of the effects of encapsulation, the pure PMMA film spectra were subtracted from the composite spectra as background. Pure PMMA thin films were characterized by PL under the same optical tune-up as the nanocomposites. The emissions in Figure 8b at 2.9 eV are common features in the PL spectra of pure PMMA. The work by Paramo *et al*., [18] reported on an emission centered at about 2.9 eV in thick slabs of pure PMMA. Additionally, the work by Nie *et al*. [15] revealed a peak at 425 nm (2.91 eV) in pure PMMA. The photoluminescence of PMMA was also observed by Morantz *et al*. [16]. This luminescence is usually assigned to unsaturated aldehyde or ketone groups undergoing *n*-π* transitions. The corrected and background subtracted spectra are presented in Figure 8c. The relative intensity of the 3.0 eV peak is substantially greater in the nanocomposite samples, which we attribute to formation of the BTO/PMMA interface. Previous work has shown [17] that coating of nanoparticles by PMMA creates carboxylate groups at the particle/polymer interface, which act as luminescent centers at 2.9 eV and may be convoluted with the bandgap emission from BTO at 3.0 eV. Indeed, it has been shown that coating of nanoparticles by polymers that do not form these carboxylate groups do not have this emission. However, we are hesitant to assign the increase of the bandgap luminescence solely to the formation of carboxylate groups at the NP surface. In the composite spectra it should be noted that the peaks observed in the pure BTO nanoparticles at 2.7 and 2.5 eV are greatly diminished while the peak at 2.2 eV is completely quenched. If carboxylate groups were solely responsible for enhancement of the peak at 3.0 eV, there is still no explanation for the quenching of the visible peaks about 2.5 eV. In similar metal oxide materials, surface hydroxyl groups act as hole traps. We suggest that these hydroxyl groups trap holes that are required for the O *2p* and Ti *3d* recombination processes that are believed to be responsible for the 2.2, 2.5, and 2.7 eV peaks.

**Figure 9.**
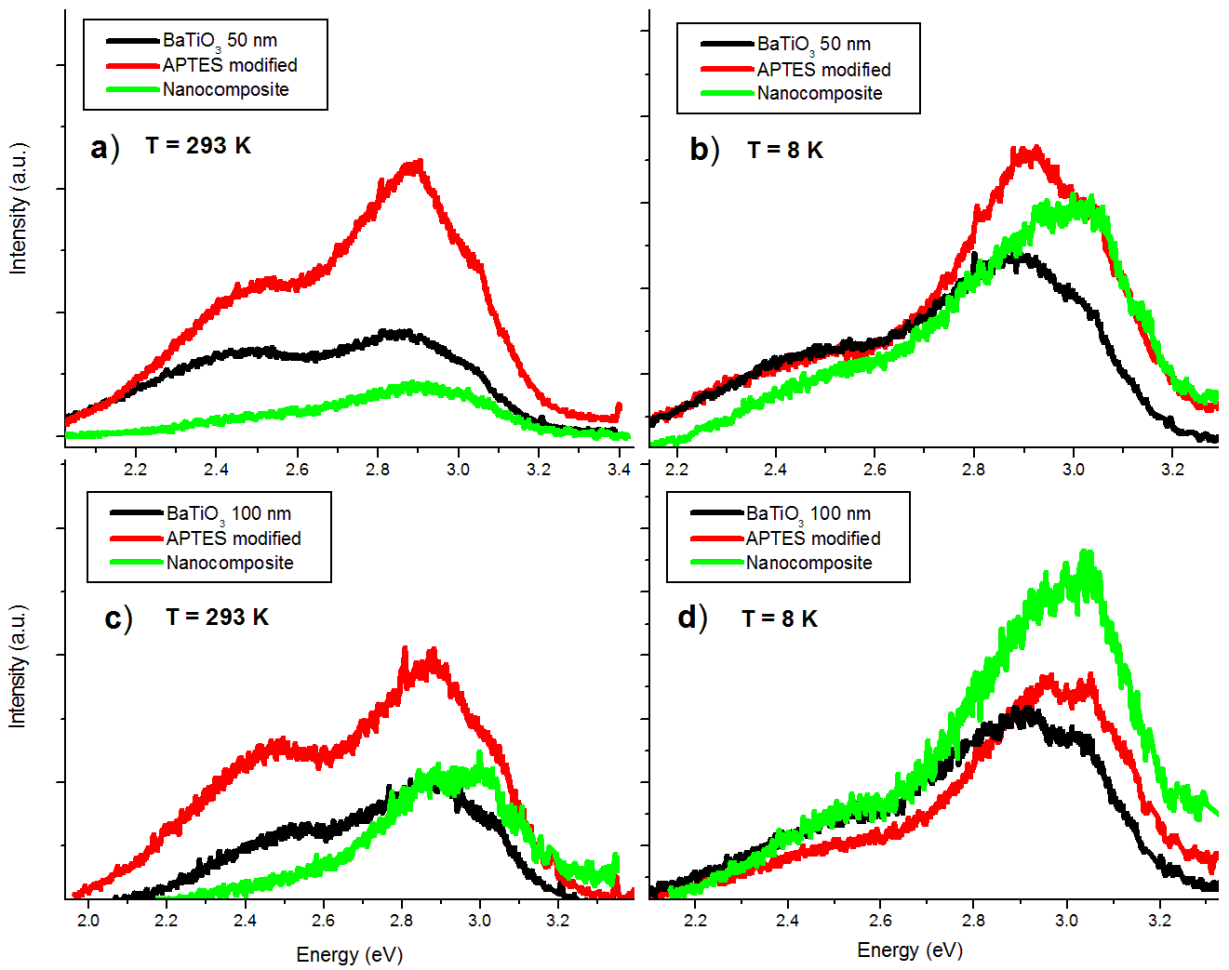
A summary of PL results: (**a**) Comparison of the 50 nm powder, APTES, and nanocomposite at RT; (**b**) Similarly, a comparison at 8 K. Note the enhanced bandgap emission from the nanocomposite; (**c**) Comparison of results from the 100 nm powder at RT; (**d**) 100 nm results at 8 K.

In the room temperature PL spectra (Figure 9a,c) it can be observed that the nanocomposite bandgap emissions are comparable to those the pure powder. The 2.5 and 2.7 eV emissions are reduced while the 2.2 eV emission is quenched completely. At low temperatures (Figure 9b,d) the bandgap emission of the nanocomposite is larger in relative intensity than both the pure powder and APTES modified nanoparticles. It appears that near-surface crystal disorder states are passivated by PMMA coating, while ATPES modification enhances overall luminescence. APTES modification is thus useful both for increasing dispersion of the nanoparticles in the polymer matrix and enhancing luminescent intensity.

**Figure 10.**
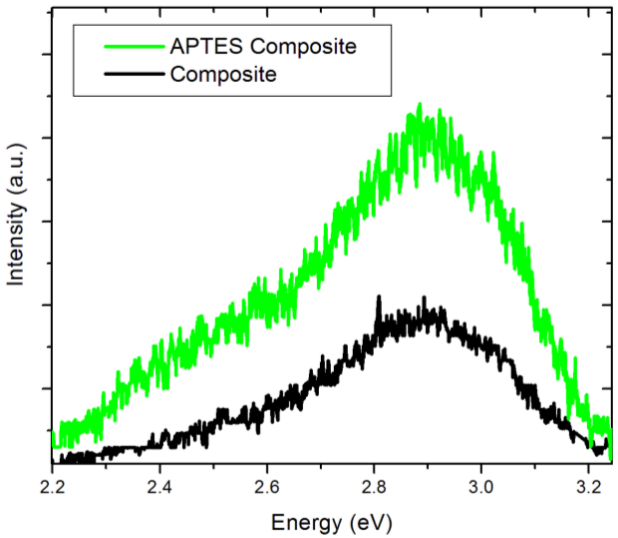
The effects of synthesizing the nanocomposite with and without the APTES modification step on the PL spectrum.

We compare the spectrum of the BTO50 APTES containing nanocomposite to the non-functionalized BTO50 nanocomposite in Figure 10. It is observed in the non-APTES containing nanocomposite that the overall luminescence is diminished when compared to the APTES nanocomposite. Also, it is interesting to note that the broad visible band is also diminished significantly. It seems it would then be possible to tune the overall luminescence and 2.5 eV emission contributions by carefully controlling the APTES coverage of the nanoparticles.

This result is useful in providing insight of the consequences of coating inorganic oxides materials. This offers potential in increasing efficiency of opticoelectronic applications of BTO/PMMA composites as well as providing an impetus to examine the effects of PMMA encapsulation on other oxide nanoparticles with surface sensitive photophysical processes. It is likely that the PMMA encapsulation causes a reduction of surface states improving the bandgap luminescence and quenching the BTO emission feature at 2.2 eV, while significantly reducing the 2.7 and 2.5 eV peaks. This interface phenomenon may be generic as a similar effect has been observed in oxide/polymer composites [17,18,20]. For ZnO, it has been proposed by [19,21] that the encapsulation of ZnO by PMMA reduces the density of surface traps, which in turn increases the concentration of near surface excitons. It is believed that a similar mechanism is in play here in BTO/PMMA composites.

## 4. Conclusions

We successfully synthesized BaTiO_3_/PMMA nanocomposite thin films using the surface functionalization solution-mixing method. FTIR was employed to confirm successful functionalization of the BTO nanoparticles by APTES. AFM has shown that APTES modification significantly reduces aggregate formation in the nanocomposite films. PL spectroscopy was used at 8 K and room temperature to quantify the effects of APTES modification and PMMA encapsulations of BTO. The relative intensities of the PL emissions at 2.7 eV and 2.5 eV that were observed in the as-received BTO nanoparticles were greatly reduced in the nanocomposite, while the emission at 2.2 eV was quenched. At the same time, the bandgap emission became significantly stronger following PMMA coating. The PMMA encapsulation reduces the density of surface traps at the BTO/PMMA interface that are associated with the visible peaks about 2.5 eV in BTO, while increasing the concentration of near-surface excitons associated with the bandgap luminescence.
